# The *Drosophila* TGF-beta/Activin-like ligands Dawdle and Myoglianin appear to modulate adult lifespan through regulation of 26S proteasome function in adult muscle

**DOI:** 10.1242/bio.029454

**Published:** 2018-04-03

**Authors:** Shaughna Langerak, Myung-Jun Kim, Hannah Lamberg, Michael Godinez, Mackenzie Main, Lindsey Winslow, Michael B. O'Connor, Changqi C. Zhu

**Affiliations:** 1 Department of Biological Sciences, Ferris State University, Big Rapids, MI 49307, USA; 2 Department of Genetics, Cell Biology and Development, University of Minnesota, Minneapolis, MN 55455, USA

**Keywords:** Activin signaling, Dawdle gene, *Drosophila*, Fruit fly, Lifespan, Myoglianin gene

## Abstract

The *Drosophila* Activin signaling pathway employs at least three separate ligands – Activin-β (Actβ), Dawdle (Daw), and Myoglianin (Myo) – to regulate several general aspects of fruit fly larval development, including cell proliferation, neuronal remodeling, and metabolism. Here we provide experimental evidence indicating that both Daw and Myo are anti-ageing factors in adult fruit flies. Knockdown of Myo or Daw in adult fruit flies reduced mean lifespan, while overexpression of either ligand in adult muscle tissues but not in adipose tissues enhanced mean lifespan. An examination of ubiquitinated protein aggregates in adult muscles revealed a strong inverse correlation between Myo- or Daw-initiated Activin signaling and the amount of ubiquitinated protein aggregates. We show that this correlation has important functional implications by demonstrating that the lifespan extension effect caused by overexpression of wild-type Daw or Myo in adult muscle tissues can be completely abrogated by knockdown of a 26S proteasome regulatory subunit Rpn1 in adult fly muscle, and that the prolonged lifespan caused by overexpression of Daw or Myo in adult muscle could be due to enhanced protein levels of the key subunits of 26S proteasome. Overall, our data suggest that Activin signaling initiated by Myo and Daw in adult *Drosophila* muscles influences lifespan, in part, by modulation of protein homeostasis through either direct or indirect regulation of the 26S proteasome levels. Since Myo is closely related to the vertebrate muscle mass regulator Myostatin (GDF8) and the Myostatin paralog GDF11, our observations may offer a new experimental model for probing the roles of GDF11/8 in ageing regulation in vertebrates.

This article has an associated First Person interview with the first author of the paper.

## Abstract (precis)

**Summary:** This article has, for the first time, demonstrated that fruit fly TGF-beta, or Activin-type ligand Daw, or Myo-initiated Activin signaling in adult fruit fly muscle tissues works as an anti-ageing factor by regulating 26S proteasome activities in those tissues.

## INTRODUCTION

Ageing is a naturally occurring body changing process that typically leads to gradual deterioration of healthy tissues and organs of the body. Various age-correlated diseases, such as cancer, diabetes, arthritis and heart disease, are significantly more prevalent among the elderly. Because of its detrimental effect on the overall human health span, acquiring detailed knowledge of the molecular and cellular mechanisms underlying the ageing process is key to providing new therapies to combat ageing and age-associated diseases. Over the last few decades, much progress has been made towards understanding how the ageing process is regulated at both cellular and molecular levels. For instance, at the molecular level the insulin/Tor signaling pathways are thought to be major components in lifespan determination across a number of animal species (Apfeld and Kenyon, 1998; Clancy et al., 2001; Holzenberger et al., 2003; Kimura et al., 1997; Ogg et al., 1997; Swindell, 2008; Tatar et al., 2001; Weindruch and Walford, 1982; Whitaker et al., 2014). In addition to the clear role of insulin signaling in regulating lifespan, other developmentally important signaling pathways have also been implicated in modulating the ageing process. Included among these are members of the TGF-β superfamily of growth and differentiation factors (Bai et al., 2013; Demontis et al., 2014; Narasimhan et al., 2011; Shaw et al., 2007), the Jun N-terminal kinase (JNK) pathway in *Drosophila* and *Caenorhabditis* *elegans* (Oh et al., 2005; Wang et al., 2005), and growth hormone pathways in mammals (Coschigano et al., 2003; Flurkey et al., 2001; Shimokawa et al., 2002), all of which seem to influence lifespan via cross talk with the insulin pathway (Brown-Borg et al., 1996; Coschigano et al., 2003; Flurkey et al., 2001; Shimokawa et al., 2002; Oh et al., 2005; Wang et al., 2005).

As animals age, cellular components such as mitochondria, cytoskeleton, and extracellular matrix undergo profound morphological and functional changes (DiLoreto and Murphy, 2015; Hansford, 1978; Sacktor and Shimada, 1972; Weinbach and Garbus, 1959). These changes at the cellular and subcellular levels are manifested either as predisposition to certain diseases or the physical decline of the body. Cellular structural alterations are only one facet of ageing cells. Loss of normal protein homeostasis in key tissues has been demonstrated by different groups to be another important hallmark of ageing (Demontis et al., 2013; Demontis and Perrimon, 2010; Tawo et al., 2017). Ubiquitination of damaged or misfolded proteins and their subsequent degradation through proteasomes is a well-conserved cellular mechanism for maintaining normal protein homeostasis in healthy tissues (Tonoki et al., 2009). Any functional impairment of proteasome-aided clearance of ubiquitinated proteins can lead to accumulation of protein aggregates in cells. These aggregates can do more harm than good to cells by affecting membrane permeability and interfering with intracellular traffic of specific molecules or vesicles (Ishihara et al., 1999; Li et al., 2001; Mandelkow et al., 2003; Relini et al., 2009).

Despite the important roles of proteasome in regulating normal protein homeostasis and thereby the ageing process, the activity of proteasome has been found to decline inevitably over time (Chondrogianni et al., 2014). The decline of the proteasome activity in specific tissues is known to be correlated with increased deterioration of the healthy status of the tissues. How this decline of proteasome function in a given adult animal species takes place over time, and what factors are responsible for the regulation of the levels of proteasome components are not fully understood. In this report, we show that in *Drosophila*, Activin signaling is an important regulator of proteasome activity in adult muscle, and that this regulation plays a positive role in lifespan determination.

In *Drosophila*, Activin signaling is initiated by the binding of any one of the three Activin-type ligands, Actβ, Daw, or Myo, to a complex of the type I transmembrane receptor Baboon (Babo) and type II transmembrane receptor Punt. Activated Babo then recruits and phosphorylates the intracellular signaling mediator, dSmad2 (also called Smox), which then associates with the co-Smad Medea protein, and the complex translocates to nucleus where it regulates target gene expression (Awasaki et al., 2011; Brummel et al., 1999; Parker et al., 2006; Serpe and O'Connor, 2006). Prior work in which Activin signaling was altered in muscle tissue suggested that Daw acts as a pro-ageing factor in part by suppressing autophagy leading to shortened adult lifespan (Bai et al., 2013). In contrast, over-expression of Myo, the *Drosophila* homolog of GDF8/11, in muscle has been reported to have both an autonomous and non-autonomous role in extending lifespan by affecting nucleolar function (Demontis et al., 2014). Similar lifespan extension effect of Myo in fruit flies has been achieved through the expression of a wild-type *Myo* gene in glial cells (Augustin et al., 2017a). The reported opposing ageing roles for Daw and Myo, which signal through a common R-Smad in *Drosophila*, together with our interest in knowing the true functions of the two ligands in regulating the tissue homeostasis of adult fruit flies prompted us to look into this issue in more detail.

In this report, we demonstrate that knocking down various *Drosophila* Activin signaling components either in the whole body or in adult muscle tends to shorten the mean life span of adult flies, while over-expression of either a wild-type *Daw* gene or a wild-type *Myo* gene (but not a wild-type *Actβ* gene) in adult skeletal muscle extends the overall mean adult lifespan. In addition, we found that this pro-survival function of Activin signaling is likely achieved through the suppression of ubiquitinated protein aggregate accumulation in muscle tissue. The suppression of ubiquitinated protein aggregates in muscle with enhanced Activin signaling appears to result in part from enhanced function of the 26S proteasomes, since knockdown of 26S proteasome subunits is able to suppress the lifespan extension effect caused by overexpression of *Daw* or *Myo* in skeletal muscle. Our results suggest that *Drosophila Daw* and *Myo* can provide protective roles in regulating adult muscle physiology and thereby the ageing process of adult fruit flies.

## RESULTS

### Assessing the expression patterns of Activin-type ligands in adult flies

**Fig. 1.**
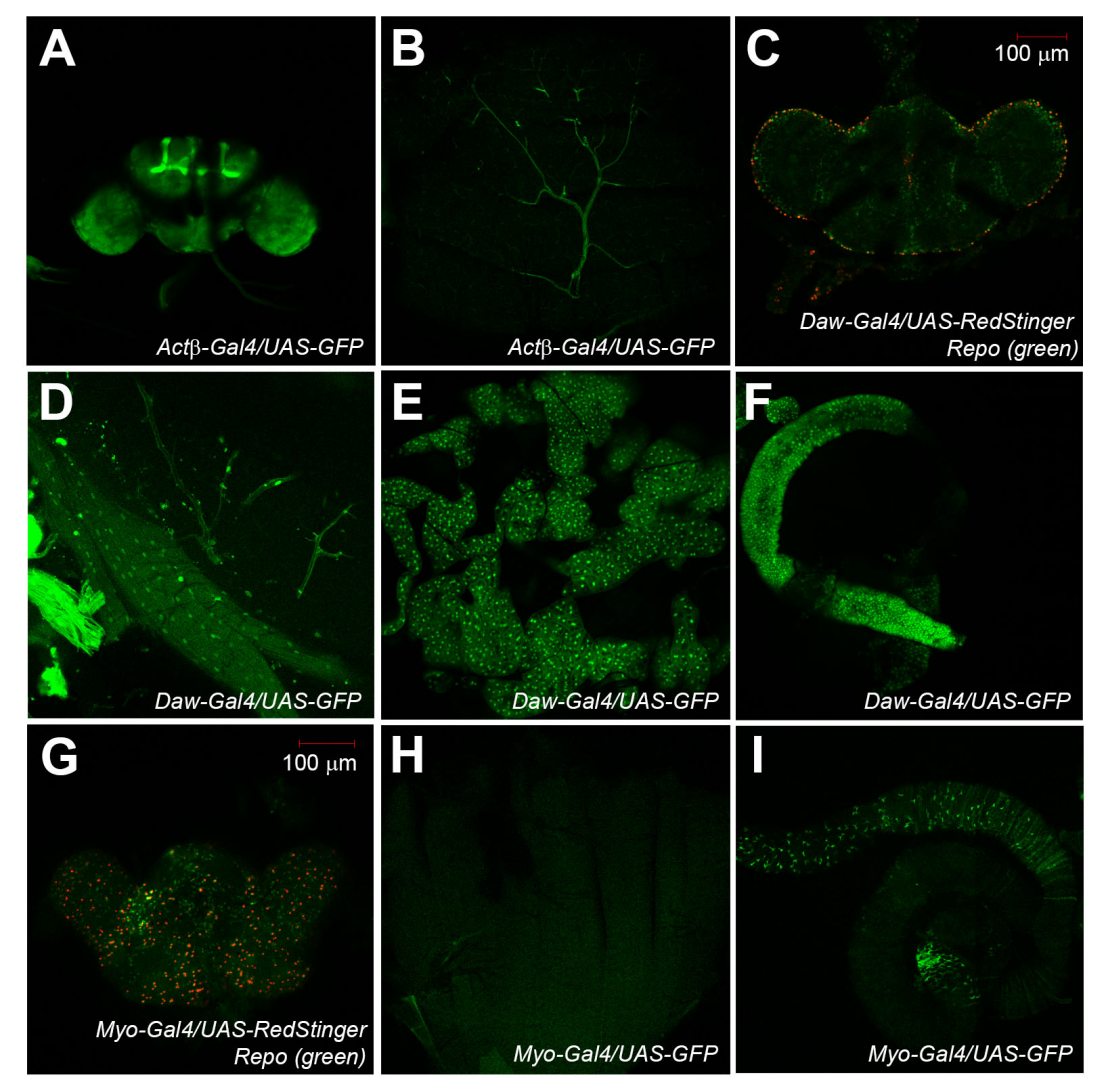
**Discrete enhancer and promoter activities of *Actβ*, *Daw*, and *Myo* genes in adult fruit flies.** Each of the three transgenic fruit fly lines, *Actβ-Gal4*, *Daw-Gal4*, and *Myo-Gal4*, was crossed either to a *UAS-GFP* reporter line or to a *UAS-RedStinger* line. The green fluorescence protein (GFP) signal or RedStinger signal from the progeny of those three crosses was monitored by a confocal microscope. The dissected adult fly brains from *Daw-Gal4/UAS-RedStinger* or *Myo-Gal4/UAS-RedStinger* were stained by an anti-Repo antibody for glial cells. (A,B) Confocal pictures showing GFP expression from brain tissues (A) and motor neurons (B) of a 1-week-old adult fruit fly with a genotype *Actβ-Gal4/UAS-GFP*. (C) Daw-expressing glial cells (red) from a 1-week-old adult fly brain that was co-stained by an anti-Repo antibody (green). (D,E,F) Strong GFP expression was observed in muscle (D), adipose tissues (E), and the gut (F) of a 1-week-old *Daw-Gal4/UAS-GFP* fruit fly. (G) *Myo-Gal4* driven RedStinger expression was observed in glial cells of adult fruit fly brain that was stained by an anti-Repo antibody (green). (H) Despite the strong expression of *Myo* in larval body wall muscle tissues, we could not see strong GFP signal from the muscle tissues of a 1-week-old *Myo-Gal4/UAS-GFP* fruit fly. (I) Strong GFP signals were also detected in the gut tissues of a 1-week-old *Myo-Gal4/UAS-GFP* fruit fly.

As a first step in analyzing the function of Activin signaling in adult flies, we examined the expression patterns of *Actβ*, *Daw*, and *Myo* in different adult tissues and organs using a transgenic approach. In these experiments, we crossed each of the three transgenic fruit fly lines, *Actβ-Gal4*, *Daw-Gal4*, and *Myo-Gal4*, that we made several years ago and have been used by a few research laboratories in their study (Awasaki, et al., 2011; Kim and O'Connor, 2014; Song et al., 2017a; Zhu et al., 2008) to a *UAS-GFP* reporter line or a *UAS-RedStinger* line. Because each of the three transgenic lines contains the enhancer and promoter DNA sequences of the gene *Actβ*, *Daw*, or *Myo* and the insulator DNA sequences, the Gal4 expression of each transgenic line just recapitulated the expression patterns of the corresponding ligands from which the enhancer and promoter DNA sequences were derived. Detailed documentation of the expression patterns of the Gal4 gene of these transgenic fruit fly lines in larval tissue has been done previously by different research groups (Awasaki, et al., 2011; Kim and O'Connor, 2014; Song et al., 2017a; Zhu et al., 2008). In our experiment described here, we mainly focused on the signals of the green fluorescence protein (GFP) or the signals produced by the RedStinger transgene from different adult tissues of the progeny from the crosses described above. When we checked the GFP or RedStinger signals representing the expression patterns of each Activin-type ligands in adult fruit flies, we found that the larval expression patterns of *Actβ*, *Daw* and *Myo* just persisted into adults with strong expression of *Actβ* in mushroom body and motor neurons and *Daw* and *Myo* in glial cells (Fig. 1A,B,C,G). Additional GFP signal for *Daw* expression pattern was also detected in adult muscle, adipose tissue, and the gut (Fig. 1D,E,F), similar to what has been reported for larva. Although strong expression of Myo has been reported in larval muscle (Parker et al., 2006; Serpe and O'Connor, 2006), the GFP signal from adult *Myo-Gal4/UAS-GFP* fruit flies is very weak in the muscle tissues of 1-week-old fruit flies (Fig. 1H). Strong Myo expression is also identified in the enterocytes of the adult gut (Fig. 1I). Because of the strong and discrete expression patterns of all three Activin-type ligands in adults, we reason that Activin signaling may play an important role in regulating adult tissue homeostasis and/or physiology in the ageing process of adult fruit flies.

### Knocking down *Drosophila* Activin signaling either in the whole body or in adult muscle shortens the average adult lifespan

To determine if Activin signaling plays a role in ageing, we sought to knockdown and overexpress various Activin signaling components specifically in adult tissues. In *Drosophila*, two different conditional methodologies have been developed to provide temporal and spatial control of gene expression. The first Gene Switch method uses a modified Gal4 activator sequence that, upon feeding the progesterone competitor mifepristone (RU486), enables activation of UAS transgenes (Roman et al., 2001). The second method employs a temperature-sensitive Gal80 (Gal80^ts^) to express Gal4 activity at high but not low temperature (McGuire et al., 2004). We tested these two systems to either knock down individual signaling components of Activin signaling pathway in adult flies using specific RNAi transgenic lines, or to enhance Activin signaling in specific adult tissues by expressing individual wild-type Activin ligands. When we tested the effectiveness of the two gene switch systems by western blotting, we found that RU486-sensitive ubiquitous Gal4 driver line *Daughterless-GS* (*DaGS*) knocked down Activin signaling when it was crossed to individual *UAS-Babo RNAi* or *UAS-Smox RNAi* lines even in the absence of RU486 treatment as measured by the levels of phosphorylated Smox proteins (P-Smox) (Fig. S1A,B), suggesting that *DaGS* line is a leaky driver line. Similar leakiness was also found from another RU-486 based ubiquitous Gal4 driver line, *β-Tubulin-GS* (*TubGS*) (data not shown). Because of the leakiness of the *TubGS* and *DaGS* transgenic lines, we switched our experiments to the Gal80^ts^-based gene switch system (McGuire et al., 2004). For these experiments, three Gal80^ts^-based transgenic lines, *Tub-Gal80^ts^, Tub-Gal4*; *Tub-Gal80^ts^, Mhc-Gal4*; and *CG-Gal4, Tub-Gal80^ts^*, were built to express different transgenes that were under the control of UAS DNA sequence either ubiquitously through *Tub-Gal4*, or in muscles by *Mhc-Gal4*, or in adipose tissue only with *CG-Gal4*. The Gal80^ts^-based gene switch system allows us to cross the three Gal80^ts^-based Gal4 driver lines to different UAS transgenic lines at 18°C where Gal4 protein will be bound by Gal80^ts^ protein so that individual transgenes under the control of UAS DNA sequences will not be expressed and normal development of fruit flies can take place. After hatching of the progeny from those crosses at 18°C, the newly hatched control and experimental male and female flies were sorted and raised at 29°C to allow the expression of individual RNAi or wild-type ligand transgenes either ubiquitously or in a tissue-specific manner. When we tested the effectiveness of this Gal80^ts^-based gene switch system in knocking down the transcripts of specific genes or in induction of the expression of any wild-type genes, we found that this system is very effective, and that it does not give leaky expression of any transgenes at low temperature 18°C (data not shown). Using this Gal80^ts^-based gene switch system, we expressed a *UAS-Babo RNAi* transgene and a *UAS-Smox RNAi* transgene either in the whole body or in muscle tissues of adult fruit flies, and found that the expression of either in adult muscles leads to reduced levels of P-Smox proteins (Fig. S1C, lanes 5 and 14 versus lanes 6 and 15, respectively). Over-expression of a wild-type *Daw* transgene in adult muscle through two independent *UAS-Daw* transgenic lines increased the levels of P-Smox about threefold (Fig. S1C, lane 10 versus lane 11) and about 93% (Fig. S1C, lane 12 versus lane 13), respectively. Over-expression of a wild-type *Myo* transgene in adult muscle increased the levels of P-Smox protein by about 12% (Fig. S1C, lane 3 versus lane 4) or 19% (Fig. S1C, lane 1 versus lane 2) while mis-expression of wild-type *Actβ* gene in adult muscle tissue was not able to enhance Activin signaling, but instead caused a 16% reduction of the signaling (Fig. S1C, lane 8 versus lane 9). The reason for this reduction of P-Smox levels by the expression of wild-type *Actβ* gene in adult muscle tissue is not quite clear yet. One noteworthy aspect of Activin signaling in adult fruit fly muscle tissues is that Activin signaling seems to be much stronger in the muscles of younger adult flies than that found in older flies as measured by the P-Smox levels from adult thoracic muscle tissues (Fig. S1C, lane 7 versus lane 16), suggesting that Activin signaling decreases over time in muscle tissues as fruit flies age. This finding is quite different from the published results of the P-Smox levels in adult fruit fly muscle tissues by Bai et al. (2013).

The western blot data suggest that our transgenic lines can produce both loss- and gain-of-function for Activin signaling in adult flies. To examine how these genetic manipulations affect ageing, we set up fruit fly crosses at 18°C, sorted the eclosed control and experimental flies at room temperature, and then shifted them to 29°C to follow the survivorship of control and experimental groups of fruit flies. Most of our transgenic fruit fly lines were created in a yellow white (*yw*) genetic background. To match the genetic background and the copy numbers of different transgenes for both our control and experimental groups of fruit flies, we crossed each of our Gal4 driver lines or our RNAi or wild-type ligand-expressing lines to a *yw* line to obtain our control fruit flies. This crossing scheme has been used by other investigators, and seems to work well for fruit fly ageing study (Wessells et al., 2004). To exclude genetic background effects on our ageing studies, we also collected another set of control and experimental fruit flies that had the same genetic makeup as those raised at 29°C and reared these fruit flies at 18°C so that none of the transgenes that were under the control of the UAS DNA sequences would be expressed. The lifespan data of our fruit flies were analyzed by using an online lifespan analysis tool called OASIS, and two independent statistical methods of Fisher's exact test and Log-rank test were employed for computing the *P*-values for assessing the differences of the lifespan of the control and experimental groups of fruit flies (Yang et al., 2011).

**Fig. 2.**
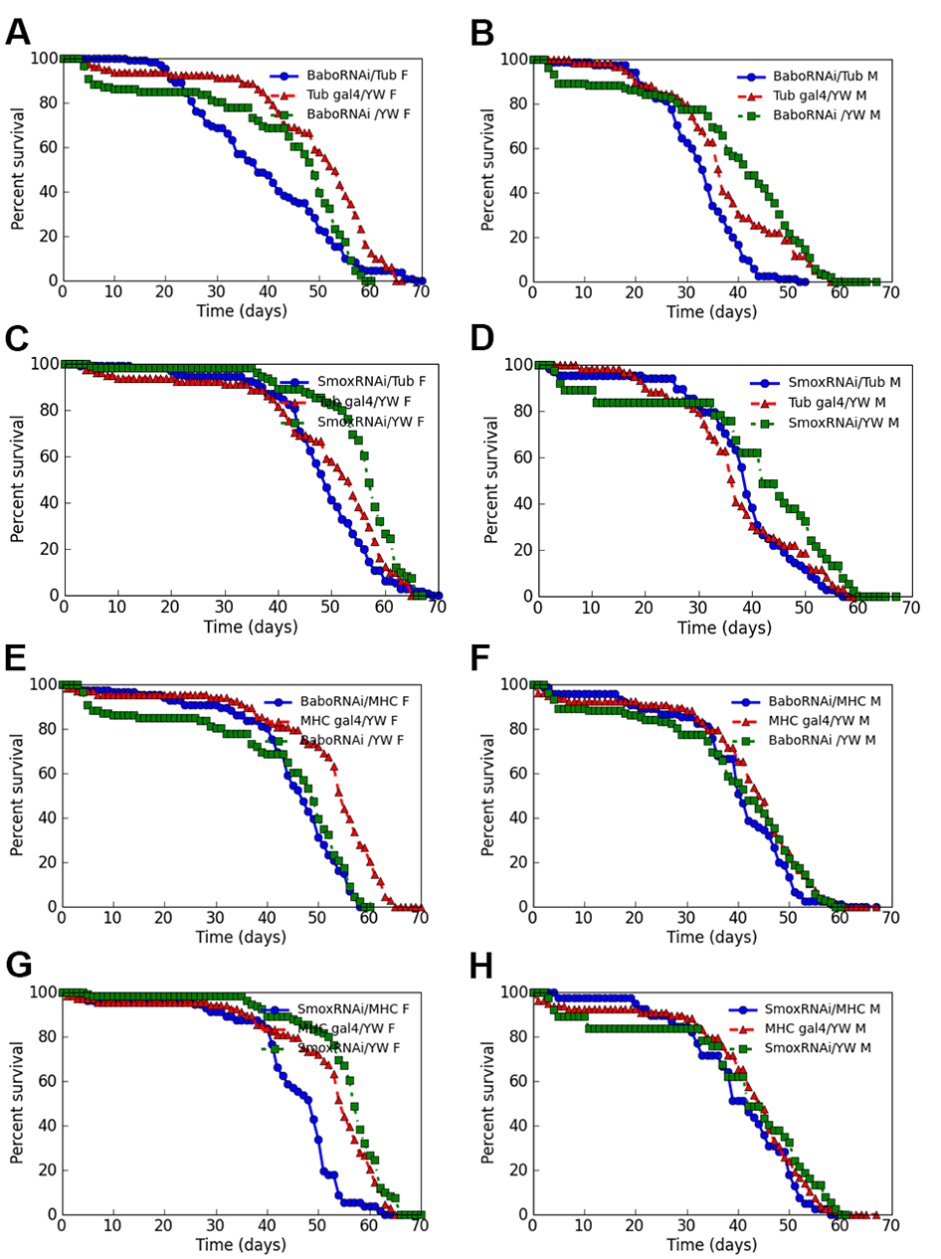
**Knocking down Babo or Smox either ubiquitously or in adult muscle tissues tends to shorten the mean life span.** (A,B) Ubiquitous expression of a *UAS-Babo RNAi* transgene through a *Tub-Gal4* driver in adult fruit flies shortened the mean life span of female and male fruit flies by about 24% or 15 days (*P*<0.05). (C,D) Ubiquitous knockdown of Smox proteins in adult fruit flies only moderately affected the ageing of female fruit flies (about 11% or 7.5 days mean life span reduction) (*P*<0.05) (C) but not the male fruit flies (D). (E,F) Muscle-specific expression of a *Babo RNAi* reduced the mean life span of these fruit flies very subtly. (G,H) Expression of a *Smox RNAi* in adult fruit fly muscle tissues resulted in a reduction of the mean life span of the female fruit flies by 6 days while for male fruit flies, the life span reduction by the expression of the *Smox RNAi* in muscle tissues is very minimum. In A to H, blue lines are survivorship curves of Babo or Smox knockdown fruit flies; red and green lines are survivorship curves of control fruit flies.

For our initial test of the effect of Activin signaling on adult *Drosophila* lifespan regulation, we crossed flies with a genotype of *Tub-Gal80^ts^, Tub-Gal4* to *UAS-Babo RNAi* transgenic animals. When the lifespan of the progeny from this cross was followed at 29°C, we found that the mean lifespan of the female and male adults expressing two copies of a *Babo RNAi* transgene ubiquitously through the *β-tubulin* promoter driven Gal4 driver (*Tub-Gal4*) is about 3 and 10 days shorter than that of the two different control fly lines (*P<*0.05) (Fig. 2A; Fig. S1D,F, Table S1). A similar life span reduction was also observed in female but not male fruit flies that ubiquitously expressed a *Smox RNAi* transgene (Fig. 2C,D; Table S1). The reason for the difference in lifespan regulation of female and male *Smox RNAi*-expressing flies is not clear. Similar sex dimorphism of lifespan regulation by other genes had been reported previously (Shen et al., 2009). Our control experiments performed at 18°C for those *Babo RNAi*-expressing fruit flies and their controls that were raised at 29°C showed that *TubGal80^ts^, TubGal4/UAS-Babo RNAi* flies did not show shortened mean or maximum lifespan when compared to their controls (*P*>0.05) (Fig. S2A), which suggests that the observed short mean lifespan of flies expressing *Babo RNAi* raised at 29°C was not likely due solely to genetic background, but was due to the ubiquitous expression of *Babo RNAi* in the adult flies.

To examine whether tissue specific knockdown of Babo or Smox affects lifespan, we started our experiments with adult muscles. Adult muscle tissues have previously been shown to play an important role in determination of *Drosophila* adult lifespan (Bai et al., 2013; Demontis and Perrimon, 2010). In addition, we found that Activin signaling is present in muscle tissues of young adult flies, but the signaling decreases quickly over time. Knockdown of Babo receptor in adult muscles resulted in almost no lifespan reduction of adult male and female fruit flies (Fig. 2E,F; Table S2), while knocking down *Smox* in adult females but not male muscle tissues caused a 6- to 10-day overall mean lifespan reduction when compared to the control flies (*P<*0.05) (Fig. 2G,H; Tables S2 and S3). Again, the sex dimorphism of the lifespan regulation by Smox is seen here, but the mechanism responsible needs further investigation. In our study, although the mean life span reduction through the expression of either a *Babo RNAi* or a *Smox RNAi* transgene showed some sex differences, there is, nevertheless, an overall trend towards a shorter lifespan when these common Activin signal transduction components are knocked down either ubiquitously in adults (Fig. 2A,B) or in muscle tissues only (Fig. 2G).

**Fig. 3.**
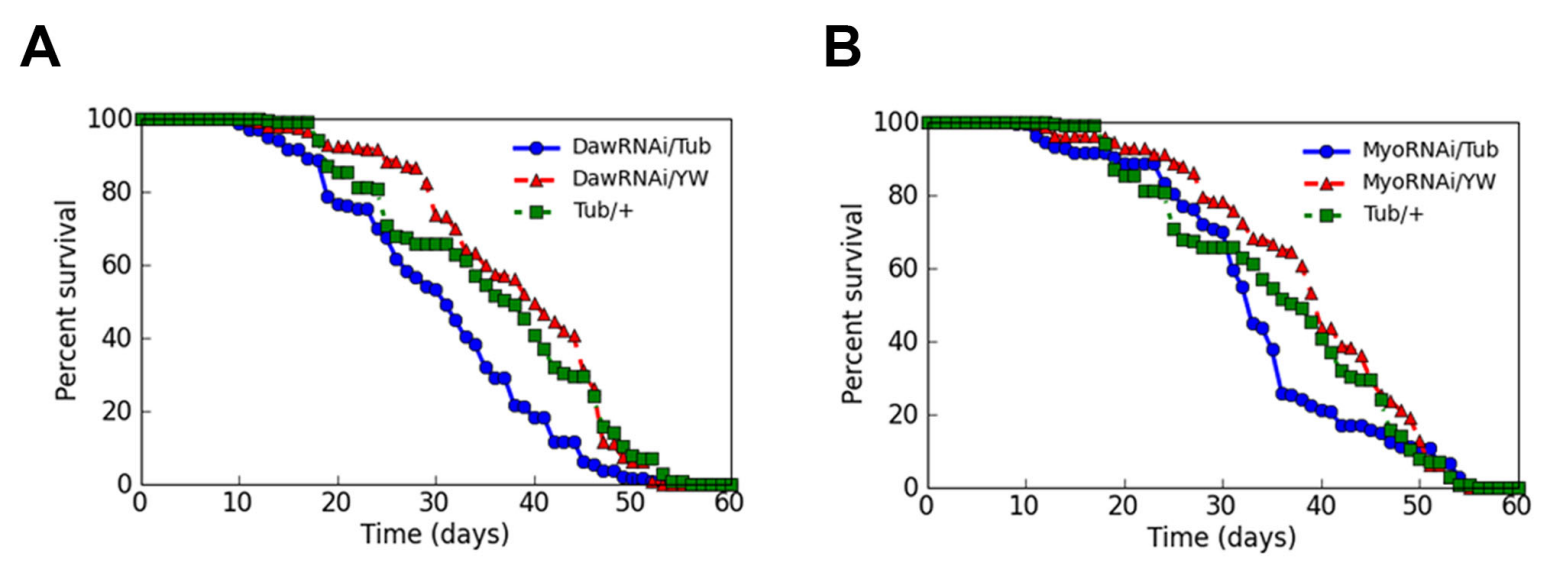
**Knocking down the transcripts of *Daw* or *Myo* gene in adult fruit flies resulted in shortened mean lifespan.** (A,B) Shortened mean lifespan was observed for the fruit flies with ubiquitous knockdown of the transcripts of *Daw* gene with a *UAS-Daw RNAi* line (Bloomington stock 34974) (*P<*0.05) (A), or for the fruit flies with ubiquitous knockdown of the transcripts of *Myo* gene with a *UAS-Myo RNAi* line (Bloomington stock 31200) (*P<*0.05) (B). In A and B, blue lines are survivorship curves for *Daw* or *Myo* knockdown fruit flies; red and green lines are survivorship curves for control fruit flies.

To test if the lifespan reduction effect caused by knockdown of *Babo or Smox* can be attributed to one or more ligand, or a combination, we also ubiquitously knocked down each of the ligands using the temperature shift strategy, and found that ubiquitous knockdown of either *Daw* or *Myo* resulted in shortened mean lifespan when compared to control flies (*P*<0.05) (Fig. 3A,B; Table S4). When the same genotype control and experimental animals in which either *Daw* or *Myo* was ubiquitous knocked down were reared at 18°C, these fruit flies did not show any significant differences in mean or maximum lifespan (*P*>0.05) (Figs S2B and S3A), again indicating that the genetic background of the *TubGal80^ts^, TubGal4/yw* and *UAS-Daw RNAi/yw* or *UAS-Myo RNAi/yw* control fruit flies and that of the experimental fruit flies *TubGal80^ts^, TubGal4/UAS-Daw RNAi* or *TubGal80^ts^, TubGal4/UAS-Myo RNAi* flies were similar, and the mean lifespan shortening effect caused by the ubiquitous expression of either a *Daw RNAi* or a *Myo RNAi* in adult fruit flies reared at 29°C is not solely due to genetic background differences. We conclude that loss of Activin signaling, at the level of ligand, receptor, or intracellular signaling mediator, consistently tends to reduce the average lifespan of adult flies.

### Over-expression of either *Daw* or *Myo* in adult muscle prolongs the mean life span

**Fig. 4.**
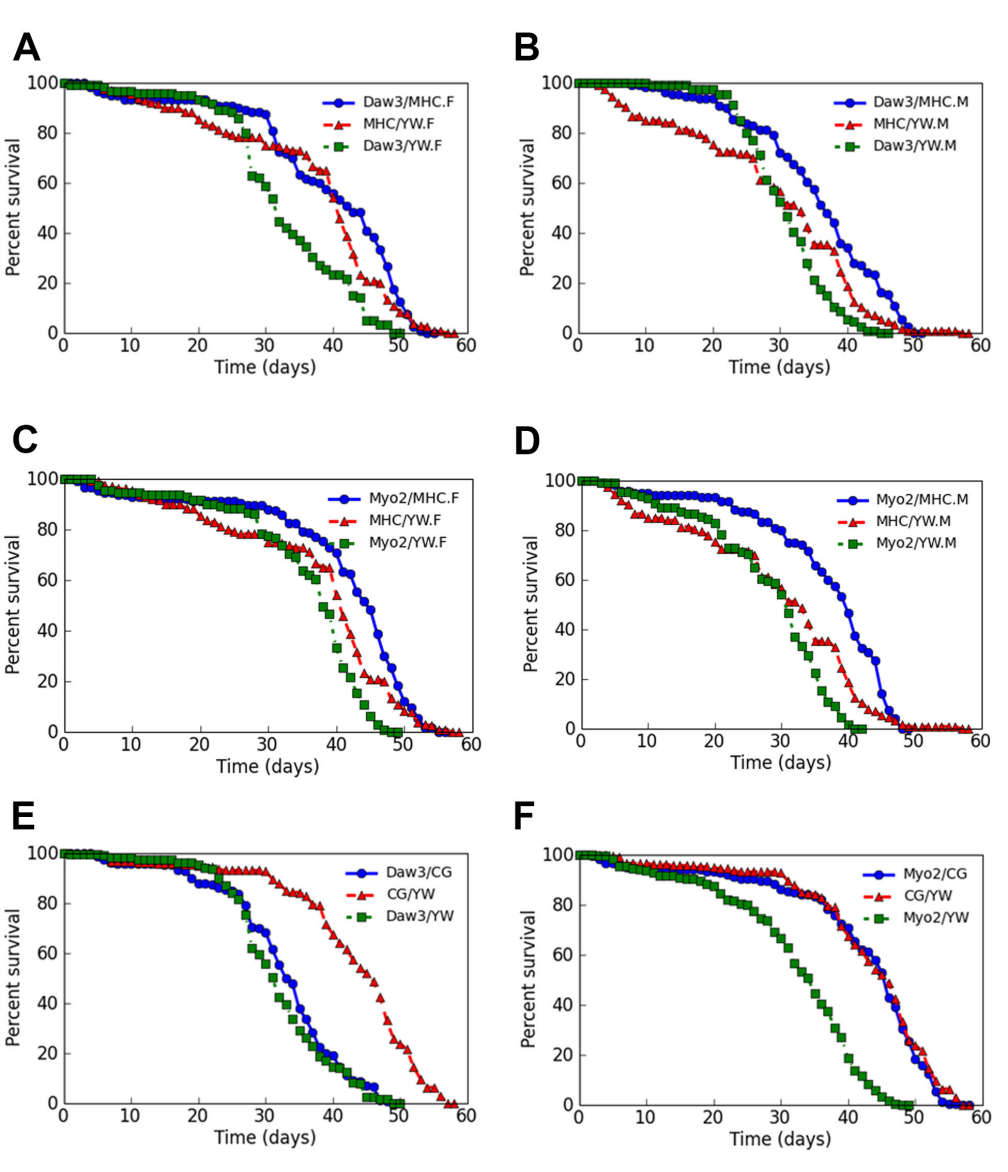
**Over-expression of either *Daw* or *Myo* in adult fly muscle but not in adult adipose tissues extends lifespan.** (A,B) Over-expression of a wild-type *Daw* transgene in adult fly muscle extended the mean lifespan by about 10% or 5 days in both female and male fruit flies (*P*<0.05). (C,D) Over-expression of a wild-type *Myo* transgene in adult fly muscle extended the mean lifespan by about 16% or 10 days (*P*<0.05) in both female and male fruit flies as well. (E) Over-expression of wild-type *Daw* in adipose tissue did not extend the overall lifespan of the experimental flies. (F) The expression of wild-type *Myo* in adult adipose tissue did not have any specific effect on lifespan. In A to F, blue curves are the survivorship curves of *Daw* or *Myo* over-expression flies; red and green curves are the survivorship curves of control flies.

Because loss of Activin signaling reduced lifespan, we examined whether gain of Activin signaling through overexpression of either *Daw* or *Myo* transgenes in adult muscle would prolong lifespan. As shown in Fig. 4A-D and Tables S5-S7, we found that over-expression of either *Daw* or *Myo* in adult muscle significantly prolonged the mean life span of both the experimental female and male flies (*P*<0.05). The mean life span extension effect of *Daw* or *Myo* overexpression was not seen when these transgenes were overexpressed in adult adipose tissue (Fig. 4E,F; Table S8). The lifespan extension effect caused by the expression of either wild-type *Daw* or *Myo* in adult fly muscle once again, appears to be independent of genetic background, since we observed no lifespan extension effect for either of the two genes when *TubGal80^ts^, Mhc-Gal4/UAS-Daw* or *TubGal80^ts^, Mhc-Gal4/UAS-Myo* flies were raised at 18°C, a condition under which *Daw* or *Myo* are not expressed (*P*>0.05) (Fig. S3B,C). In summary, our gain-of-function data for Daw or Myo are consistent with the idea that both ligands can act as anti-ageing factors when expressed in *Drosophila* adult muscle.

### Activin signaling is required for regulating protein homeostasis in adult muscle

Our experimental data suggest a role in skeletal muscle for Activin signaling in regulation of adult *Drosophila* lifespan. A number of *Drosophila* studies have implicated protein homeostasis as an important contributing factor in modulating the ageing response (Bai et al., 2013; Demontis and Perrimon, 2010; Hunt and Demontis, 2013). To test if *Drosophila* Activin signaling modulates the ageing process through regulation of protein homeostasis in adult muscle, we examined the levels of ubiquitinated protein aggregates in the thoracic indirect flight muscles of both control and test animals using immunohistochemistry.

**Fig. 5.**
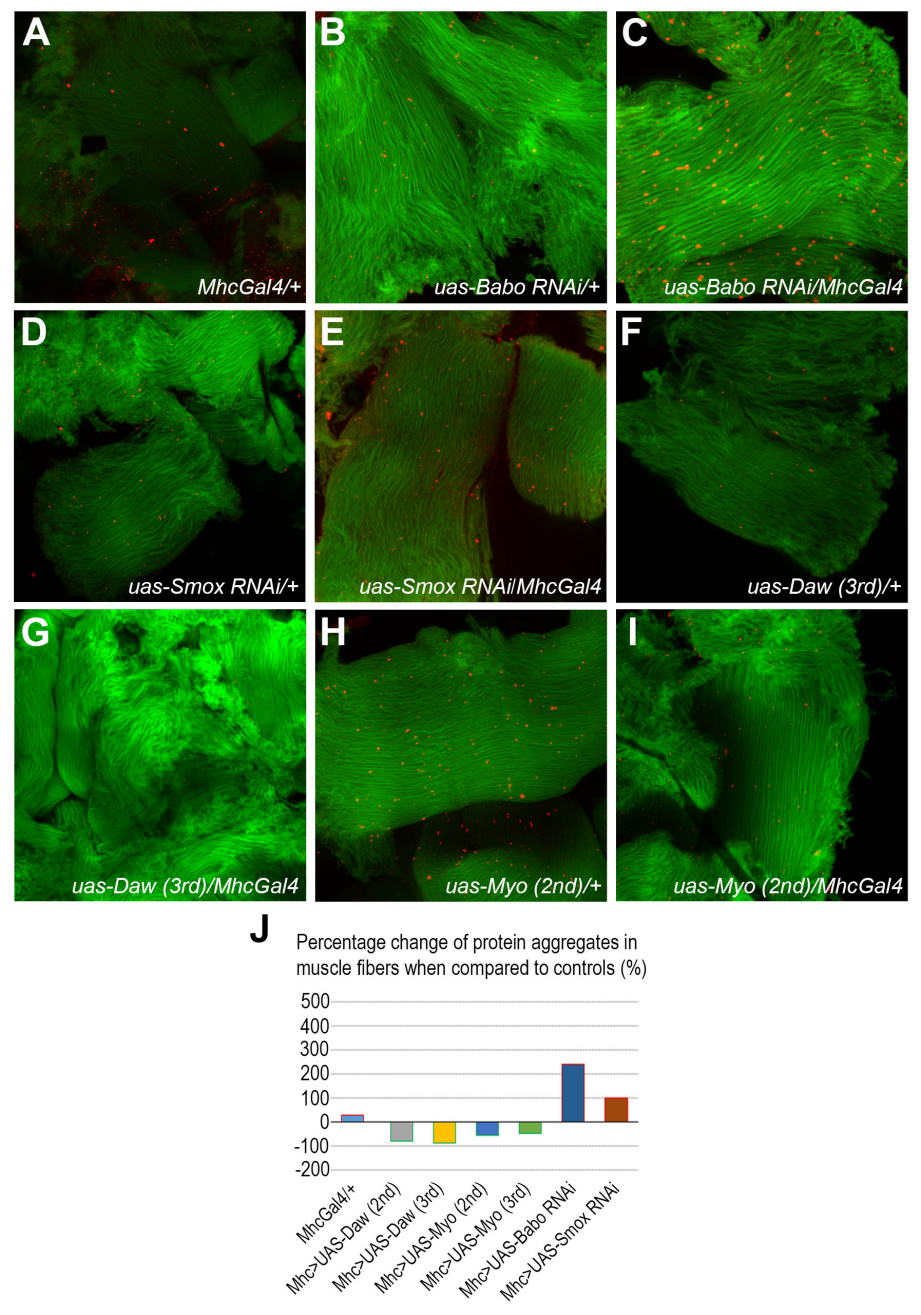
**Fruit fly Activin signaling regulates normal protein homeostasis in adult fruit fly muscle tissues.** (A-I) Thoracic muscle tissues of 20-day-old female fruit flies of different genotypes were stained by an anti-mono/polyubiquitin antibody (FK2) for ubiquitinated protein aggregates (red) and counter stained by CytoPainter Phalloidin-iFluor 488 for F-actin of muscle fibers (green). Intermediate levels of ubiquitinated protein aggregates (red dots) were observed from thoracic muscle of *MhcGal4/+* control fruit flies (A), *UAS-Babo RNAi/+* control fruit flies (B), *UAS-Smox RNAi/+* control fruit flies (D), *UAS-Daw (3rd)/+* control fruit flies (F), and *UAS-Myo (2nd)/+* control fruit flies (H). Expression of a *Babo RNAi* transgene (*UAS-Babo RNAi)* or a *Smox RNAi* transgene (*UAS-Smox RNAi)* in adult thoracic muscle tissues with a *MhcGal4* driver resulted in elevated levels of protein aggregates as shown in C and E, respectively, when compared to those of the control tissues (*P*<0.001). Expression of a wild-type *Daw* transgene (*UAS-Daw *on the third chromosome**) or a wild-type *Myo* transgene (*UAS-Myo *on the second chromosome**) in adult fly muscle effectively suppressed the appearance of ubiquitinated protein aggregates as shown in G and I, respectively, when each of the tissue samples from G and I was compared to their respective controls from F and H (*P*<0.001). (J) Quantified relative levels of the ubiquitinated protein aggregates from the thoracic muscle tissues of different genotypes of fruit flies from A to I.

Consistent with previously published results (Demontis and Perrimon, 2010; Hunt and Demontis, 2013), we find that mono- or poly-ubiquitinated protein aggregates are barely detectable in newly hatched adult muscle tissues, but they begin to accumulate at about day 5 post hatching (data not shown) and are readily observed in 20-day-old flies. When we compared the levels of the protein aggregates present in control 20-day-old muscle (Fig. 5A,B,D) to those of the experimental flies that expressed either *Babo RNAi* (Fig. 5C; Fig. S2E versus C and D) or *Smox RNAi* (Fig. 5E) in adult muscle, we found that knockdown of Activin signaling significantly increased the number of ubiquitinated protein aggregates observed in those tissues (*P*<0.05) (Fig. 5C versus A and B, Fig. 5E versus D and A). In contrast, overexpression of either *Daw* or *Myo* in muscle significantly suppressed the appearance of the protein aggregates (*P*<0.05) (Fig. 5G versus F, Fig. 5I versus H and J; Fig. S2G versus C and F, Fig. S2I versus C and H, Fig. S2K versus C and J). Taken together, these data suggest that Activin signaling is required for maintaining proper protein homeostasis in *Drosophila* adult muscle.

### Activin signaling does not appear to modulate protein homeostasis in adult muscle through up-regulation of autophagy

**Fig. 6.**
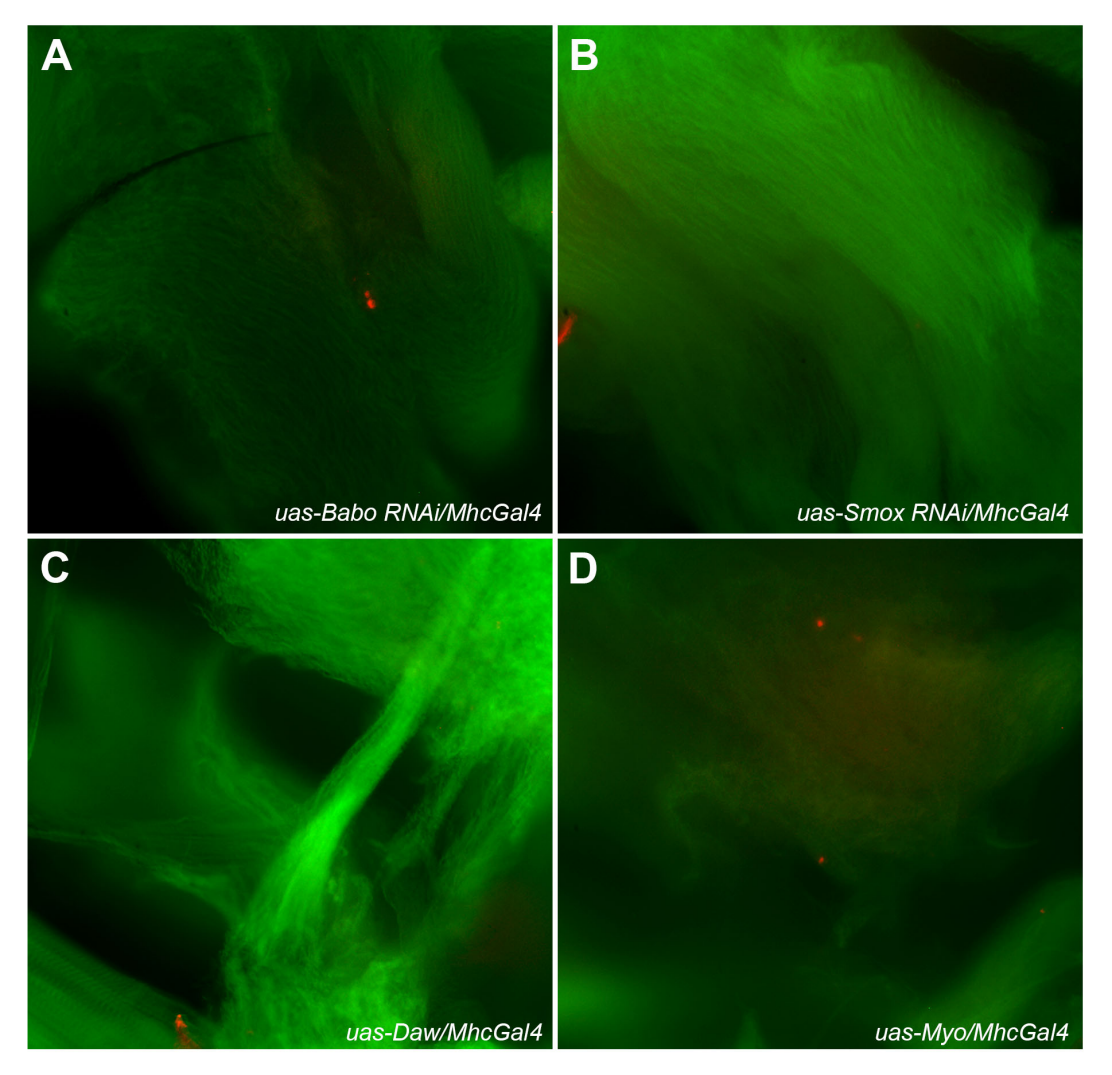
**Alteration of the levels of Activin signaling in adult fruit fly thoracic muscle did not change the overall autophagy activities in those tissues.** (A-D) Thoracic muscle tissues of 19-day old female fruit flies of four different genotypes were imaged for autophagosomes labeled by Lysotracker (red) and for F-actin of muscle fibers stained by CytoPainter Phalloidin-iFluor 488 (green). (A) Babo receptor knockdown muscle tissues. (B) Smox knockdown muscle tissues. (C) Daw over-expression muscle tissues. (D) Myo over-expression muscle tissues. No significant difference of autophagosomes from the thoracic muscle tissues of the four different genotypes of fruit flies was seen (*P*>0.05).

**Fig. 7.**
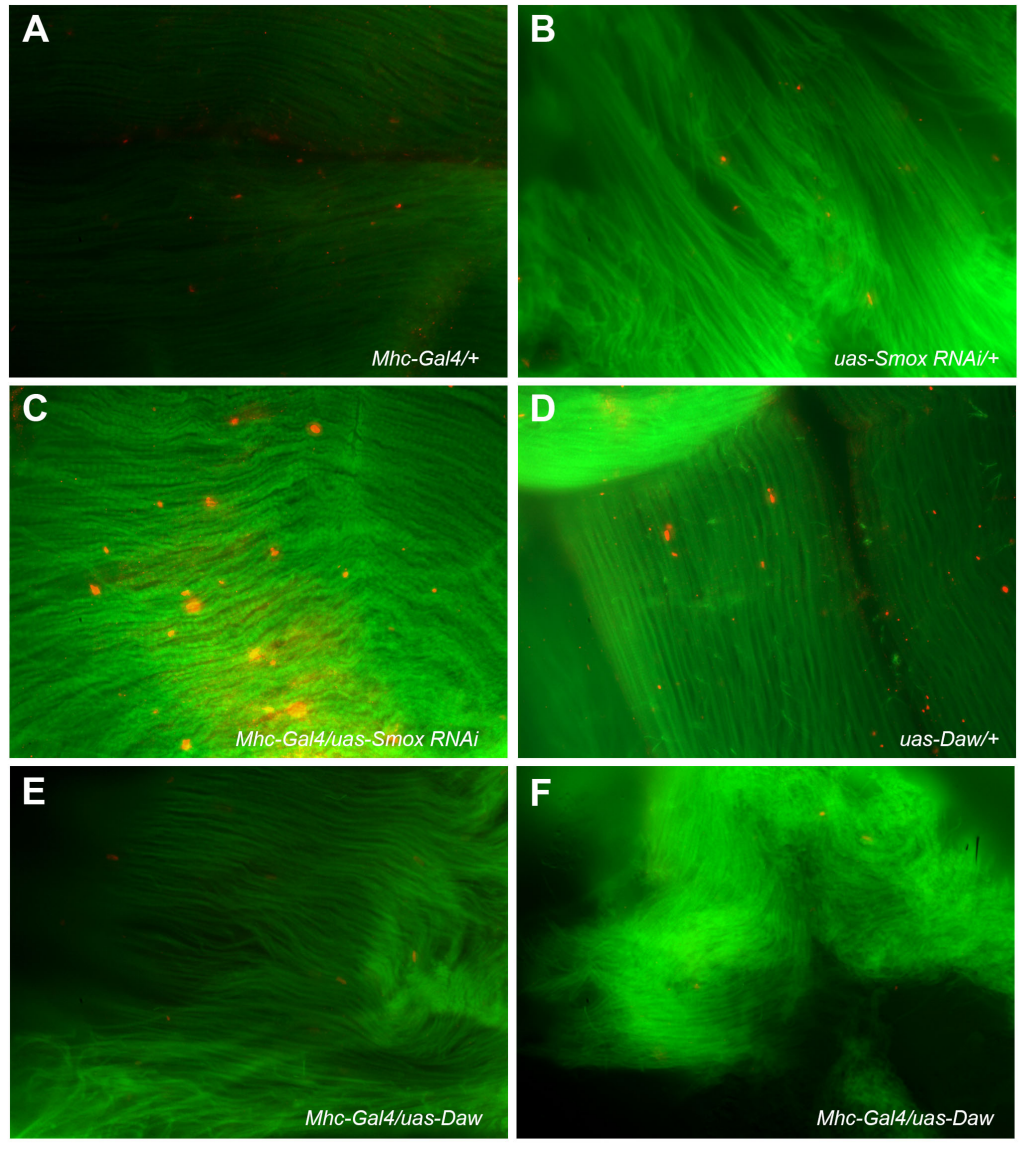
**Starvation-induced autophagy activities do not alter the levels of ubiquitinated protein aggregates in our control and experimental fruit flies.** (A-F) FK2 antibody staining results for ubiquitinated protein aggregates (red dots) from the thoracic muscle tissues of 20-day-old female control (A,B,D) and experimental fruit flies (C,E,F) that were starved for 24 h before the staining was done. All muscle tissues for this panel were counter stained with CytoPainter Phalloidin-iFluor 488 (green). Note that starvation itself did not alter the levels of ubiquitinated protein aggregates in thoracic muscle fibers of different genotypes of fruit flies (A-F) when they were compared to those of non-starved fruit flies of the same genotypes as shown in Fig. 5. High levels of protein aggregates are still seen in thoracic muscle of Smox knockdown fruit flies (*Mhc-Gal4/UAS-Smox RNAi)* (C). Starvation did not change the reduced levels of ubiquitinated protein aggregates caused by over-expression of Daw in fly muscle tissues (E,F) either.

Protein homeostasis is maintained by at least two different cellular mechanisms: autophagy and 26S proteasome activity (Urushitani et al., 2010). Autophagy helps degrade and recycle macromolecules and different organelles in response to various types of stress (Kaminskyy and Zhivotovsky, 2012; Mizushima, 2011; Vellai, 2009). To determine if autophagy is part of the cellular mechanism involved in mediating Activin's effect on protein degradation, we examined autophagic puncta in adult thoracic muscle from either control or experimental flies using either lysotracker or a lysosome-associated membrane protein 1 (LAMP1) antibody (data not shown). Using the Lysotracker staining approach, we observed no difference in the levels of autophagosomes in adult thoracic muscles of the flies that expressed either a *Babo RNAi* or a *Smox RNAi* transgene or wild-type *Daw* or *Myo* in muscle (*P>*0.05) (Fig. 6A-D). To further assess whether autophagy is either not involved, or minimally involved in mediating Activin signaling-regulated ubiquitinated protein degradation, we starved 19-day-old control and experimental flies that expressed either a *UAS-Babo RNAi* transgene or a *UAS-Smox RNAi* transgene or wild-type *Daw* or *Myo* gene in adult muscle for 24 h and examined the ubiquitinated protein aggregates thereafter. Our data show that starvation itself did not alter the levels of the protein aggregates in the adult muscle tissues of both the control and experimental flies (Fig. 7A-F; Fig. S3D). The levels of ubiquitinated protein aggregates in starved *Smox RNAi* expression flies remained high compared to those from the muscle of the control flies (Fig. 7C versus A and B). The ubiquitinated protein aggregates from the muscle of starved wild-type *Daw*-expressing flies also remained low when compared to the controls (Fig. 7E and F versus A and D). Based on these data, we conclude that the alteration of ubiquinated proteins due to either gain or loss of Activin signaling in adult fly muscle is independent of autophagic activity.

### Activin signaling directly or indirectly alters 26S proteasome activity in adult muscles

**Fig. 8.**
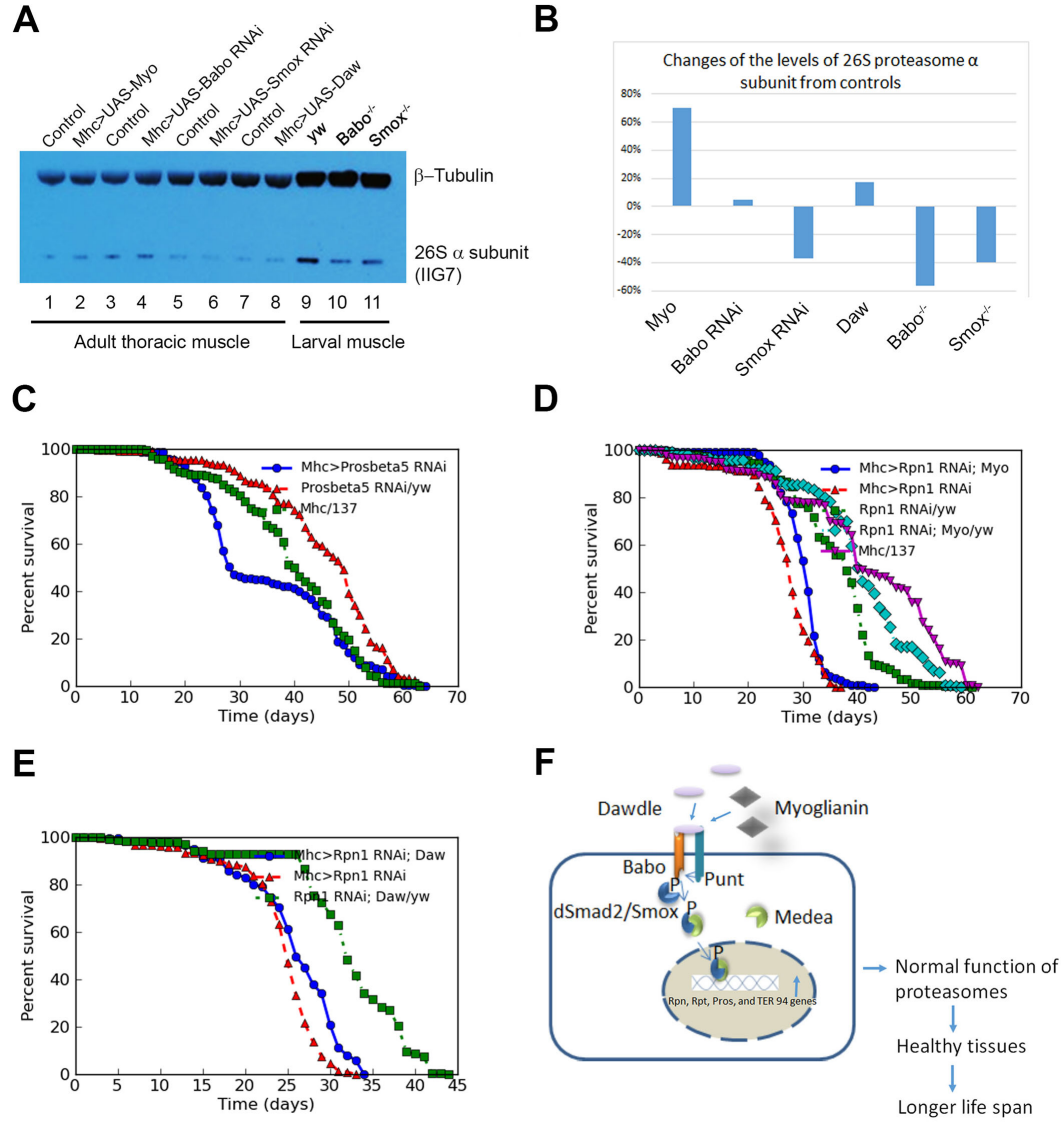
**Activin signaling regulates the life span of adult fruit flies by modulating the function of proteasome in adult fruit fly muscle tissues.** (A) Western blot data for the protein levels of a 26S proteasome catalytic α subunit from 1-week-old adult thoracic muscle and larval body wall muscle of different genotype fruit flies. (B) Quantification results of the western blot data from A. (C) Knocking down the transcripts of *Prosβ5* gene in adult fruit fly muscle with a *UAS-Pros β5 RNAi* line (Bloomington stock number 53974) and a *TubGal80^ts^; MhcGal4* driver line resulted in shortened mean lifespan of the experimental fruit flies when compared to the control fruit flies (*P*<0.05). Blue curve: survivorship of *Mhc-Gal4/UAS-Prosβ5 RNAi* flies. Red curve: survivorship of *UAS- Prosβ5 RNAi/yw* control flies. Green curve: survivorship of *Mhc-Gal4/v^1^* control flies. (D) Co-expression of a proteasome RNAi transgene *UAS-Rpn1 RNAi* together with wild-type *Myo* in adult muscle has abolished the lifespan extension effect of *Myo.* The lifespan extension effect of Myo shown in Fig. 4C and D is not seen any more here when both *UAS-Rpn1 RNAi* and *UAS-Myo* were activated by MhcGal4 in adult muscle tissues (blue curve versus green, cyan, and dark purple curves). Green curve: survivorship of *UAS-Rpn1 RNAi/yw* flies. Cyan curve: survivorship of *UAS-Rpn1 RNAi, UAS-Myo/yw* flies. Dark purple curve: survivorship of *MhcGal4/137* control flies. (E) Expression of an *Rpn1 RNAi* in adult fruit fly muscle severely compromised the lifespan extension effect by the over-expression of a wild-type *Daw* gene in adult muscle (blue curve versus the blue curves in Fig. 7A and B). Blue curve: survivorship of *TubGal80^ts^, Mhc-Gal4/UAS-Rpn1 RNAi, UAS-Daw* flies. Red curve: survivorship of *TubGal80^ts^, Mhc-Gal4/UAS-Rpn1 RNAi* flies. Green curve: survivorship of *UAS-Rpn1 RNAi, UAS-Daw/yw* flies. The survivorship of the control *TubGal80^ts^, Mhc-Gal4/137* flies is not shown here, but can be seen as the magenta curve (*Mhc/137*) in D. (F) A model for the function of fruit fly Activin signaling in lifespan regulation through adult muscle tissues. In fruit fly muscle tissues, Activin signaling works as an anti-ageing factor, which up-regulates the major protein subunits of proteasomes so that any damaged or misfolded proteins can be cleared fast enough to ensure healthy status of the tissue. As a result of this regulation, fruit flies can achieve a longer healthy lifespan.

Since autophagy does not seem to be the major mechanism by which of Activin signaling mediates protein homeostasis in muscle, we next examined if Activin signaling alters 26S proteasome function in adult muscle. To determine if Activin signaling affects proteasome levels, we performed a western blot using a monoclonal antibody (IIG7), which is known to detect a catalytic subunit of the 26S proteasome. As shown in Fig. 8A and B, the protein level of the proteasome α subunit detected by antibody IIG7 was increased by about 70% in the muscle tissues of adult fruit flies that expressed a wild-type *Myo* transgene in those tissues (lane 2 versus lane 1). A similar but lower induction (around 18%) of the same proteasome α subunit was also observed from adult muscle of the flies that expressed a wild-type *Daw* gene in muscle (lane 8 versus lane 7). Consistent with the gain-of-function data, our loss-of-function data show that the protein levels of the affected proteasome subunit were reduced by about 37% in the thoracic muscle tissues of the fruit flies that expressed a *Smox RNAi* transgene in those tissues (lane 6 versus lane 5) though no reduction of the proteasome subunit was observed from thoracic muscle of *babo* knockdown flies (lane 4 versus lane 3), which could be due to experimental error, or due to ineffective knockdown of Babo receptor by the RNAi transgene. Similar to the results obtained from adult fly muscle, *Babo* or *Smox* mutant larval body wall muscle also exhibited low levels of the proteasome α subunit (lanes 10 and 11 versus lane 9). The larval body wall muscle of *Babo* and *Smox* mutants displayed about 55% and 40% reduction of the proteasome subunit, respectively, when compared to that of the control sample. Taken together, our data suggest that Activin signaling, initiated by either Daw or Myo, serves as a positive regulator for the proteasome components in adult muscle.

To further test if 26S proteasome activity is functionally connected to Activin signal-regulated ageing, we examined if expressing a particular proteasome RNAi transgene in adult muscle affects adult lifespan. We found that RNAi knockdown of the *Prosβ5* gene, which encodes a proteasome subunit, in adult muscle reduced the average lifespan precipitously at about the time of 25% lethality when compared to controls (*P*<0.05) (Fig. 8C). The lifespan reduction caused by the expression of the *UAS-Prosβ5 RNAi* is correlated with increased levels of ubiquitinated protein aggregates in muscle tissues (Fig. S4A,B). A similar lifespan shortening effect has been observed in flies expressing a *Prosα6 RNAi* transgene in adult muscle (data not shown). To test if knocking down individual proteasome components in adult fly muscle can compromise the *Daw* or *Myo* overexpression-induced lifespan extension effect, we built a *UAS-Myo* or a *UAS-Daw* transgene and a *UAS-Rpn1 RNAi* transgene into the same fly line and expressed these transgenes together in adult fly muscle, and the survivorship of these flies was monitored and compared (Fig. 8D,E; Table S9). The wild-type *Rpn1* gene encodes a regulatory subunit of the proteasome. We find that expression of an *Rpn1 RNAi* alone in adult muscle resulted in a severe lifespan reduction when it was compared to the lifespan of the control flies (Fig. 8D,E; Table S9). Importantly we further find that the lifespan reduction effect caused by knockdown of the transcripts of *Rpn1* gene in adult fly muscle completely abolished the lifespan extension effect caused by the over-expression of a wild type *Daw* or *Myo* gene in muscle (blue curves in Fig. 8D and E versus the blue curves in Fig. 4A-D; Table S9). These data suggest that proteasome-induced degradation of misfolded or damaged proteins in adult fly muscle plays an essential role in mediating the Daw- and Myo-initiated lifespan-extension effect in muscle.

## DISCUSSION

Previous studies by several groups have demonstrated important roles for Activin signaling in regulation of *Drosophila* larval and pupa development, strength of synaptic transmission, and cellular metabolism (Augustin et al., 2017b; Awasaki et al., 2011; Brummel et al., 1999; Childs et al., 1993; Das et al., 1999; Nakao et al., 1997; Chng et al., 2014; Ghosh and O'Connor, 2014; Gibbens et al., 2011; Kim and O'Connor, 2014; Lo and Frasch, 1999; Parker et al., 2006; Serpe and O'Connor, 2006; Song et al., 2017a,b; Ting et al., 2007; Zheng et al., 2003; Zhu et al., 2008). Here we show that Activin signaling also regulates average *Drosophila* lifespan in part through the modulation of protein homeostasis in adult muscle. In our hands, reducing general Activin signaling by knockdown of Smox or Babo shortened average lifespan, while overexpression of either of the two ligands Daw or Myo in adult fly muscle extended average lifespan. Thus, Activin signaling may act as an ageing antagonist at least in muscle. However, it is important to recognize that tissue-specific effects on general ageing of the whole organism might be quite different depending on which tissue is targeted, or when compared to effects elicited by general knockdown in all tissues. This is of special relevance to Activin signaling, at least for the ligand Daw, which has been shown to be a systemic factor that regulates multiple metabolic and physiologic properties in different tissues (Ghosh and O'Connor, 2014; Chng et al., 2014). Therefore, one must be cautious not to extrapolate a single tissue knockdown result as signifying the only role for how a systemic factor such as Activin might modulate a complex phenomenon such as ageing.

While our results are internally consistent in that both ligands are thought to signal through the common transducer Smox (Awasaki et al., 2011; Serpe and O'Connor, 2006; Parker et al., 2006), and therefore loss and gain of function of either ligand might be expected to give similar phenotypes, they do not entirely agree with previous reports. On the one hand, our findings are in agreement with those published by Demontis et al. (2014), where Myo overexpression prolonged lifespan, but differ somewhat from those reported by Augustin et al. (2017a). This group found elevated Myo expression in glial cells prolonged lifespan, but they did not observe a lifespan extension effect when Myo was expressed in muscle. The discrepancy between the results reported by Augustin et al. (2017a) and those described by Demontis et al. (2014) and ourselves might be due to use of different muscle-‘specific’ Gal4 lines. We have observed that Mhc-Gal4 is a very strong Gal4 driver in adult muscle tissues while Mef2-Gal4 is not. In addition, Mef-2 Gal4 shows expression in some neurons while Mhc does not. These differences in expression levels and cell types could explain the discrepancy and highlight the importance of careful consideration of Gal4 expression patterns when comparing results between different groups.

In terms of the Daw effect on ageing, Bai et al. (2013) reported that knockdown of *Daw*, *Babo* or *Smox* increased autophagy in muscles and thereby extended average lifespan. When we tested if Activin signaling is an upstream regulator for autophagy activities in adult fly muscle, we did not note any difference in the number of autophagosomes observed in the adult muscle of Babo or Smox knockdown flies and Daw or Myo over-expression flies, suggesting that autophagy may not be the central mechanism involved in Activin signal-regulated lifespan determination. In addition, when we knocked down the transcripts of different Atg genes, such as Atg1 and Atg8a in adult muscle, we did not observe any obvious lifespan shortening effects (data not shown). At present, it is unclear as to the cause of this discrepancy. As described above, one might expect that the two ligands, Daw and Myo, which signal through the same set of receptors and a common downstream set of signal transducing components would produce a similar phenotype in response to genetic manipulations with perhaps just a change in the intensity of the response, but not a complete switch in the direction of the response i.e. anti-ageing as opposed to pro-ageing when the signal is lost.

One possible explanation for the discrepancy between our observations and those of Bai et al. (2013) could be due to the utilization of two different conditional gene expression systems. As we report here, although RU486-based gene switch system is particularly useful for allaying genetic background concerns, it also has its own drawbacks. In particular, leaky expression of the Gal4 driver in the absence of RU486 treatment, as we found in this study, and also reported by others (Scialo et al., 2016) can be a significant problem when weaker Gal4 driver lines are used. In addition, it is known that the feeding behavior of *Drosophila* can be affected by RU486 treatment (Yamada et al., 2017), which could further complicate interpretation of the lifespan studies using this system.

Given the potential problems with the RU486-based gene switch methodology (Scialo et al., 2016; Yamada et al., 2017) and a central concern of genetic background effects in ageing study (Partridge and Gems, 2007), we instead employed the Gal80^ts^ gene switch system to control the expression of specific transgenes in adult flies. This method enabled us to culture both control and experimental groups of fruit flies at two different temperatures, i.e. 18°C versus 29°C. At 18°C, because of the binding of the temperature-sensitive Gal80^ts^ protein to Gal4 protein, none of the transgenes under the control of UAS DNA sequences would be expressed, and the ageing profile of these fruit flies can be well recorded and used for monitoring any potential genetic background effects for the fruit flies with the same genetic makeup raised at 29°C, a temperature at which individual transgenes under the UAS enhance sequences are transcribed because of inactivation of Gal80^ts^ and the activation of Gal4 protein. We acknowledge that this is not a perfect but a valid methodology. To minimize any genetic background effect on ageing study, back-crossing all transgenic fruit flies to a wild-type fly line multiple times has been suggested and adopted by a number of research labs. In our opinion, back-crossing individual fruit fly lines to a wild-type line may help study the genetic effect on the absolute average or maximum lifespan of individual population of fruit flies, but it may not solve the subtle genetic differences of the two parental lines used for the expression of individual transgenes and the genetic differences of the parental lines and the progeny for lifespan observation. From a technique point of view, back-crossing the various compound transgenic lines to a wild-type fruit fly line required for these studies also poses a significant technical challenge. Based on the current available technology in fruit fly research community, we think that the Gal80^ts^ approach seems to be a reasonable compromise as long as its limitations and strengths for interpreting and comparing the experimental results are carefully monitored and considered.

Our finding that Activin signaling either directly or indirectly regulates proteasome function is consistent with previous reports suggesting that protein homeostasis is an important determinant of the ageing response (Chondrogianni et al., 2014). The main role of proteasomes is to regulate protein homeostasis through the degradation of misfolded or damaged proteins following ubiquitination. Any deregulation of the levels or function of the proteasome can lead to either accelerated or delayed ageing (Keith et al., 2016; Tonoki et al., 2009; Tsakiri et al., 2013). In *Drosophila*, individual proteasome components are known to decrease over time as flies age (Chondrogianni et al., 2014). The reason for this decline is not clear. In our study, we found that Activin signaling is a positive regulator of several proteasome components, and that Activin signaling goes down quickly as fruit flies age. When we over-expressed wild-type *Daw* or wild-type *Myo* gene in adult fly muscle, we see an increase in the levels of certain proteasome components and prolonged mean lifespan of adult fruit flies. These data suggest that Activin signaling is a positive regulator of at least some proteasome components in adult fly muscle. Activin signaling is likely not the only upstream positive regulator of individual proteasome components since several subunits have been shown to be up-regulated by Nrf2 in *Drosophila* (Tsakiri et al., 2013) and by EGF signaling in *C. elegans* (Liu et al., 2011). It will be important to determine in future studies if there is any genetic interaction of Activin signaling with these factors in regulating protein homeostasis through modulation of proteasome activity.

Although autophagy is another known cellular mechanism for maintaining protein homeostasis in different organisms and in different tissue types (Kim et al., 2015), and it has been implicated in ageing regulation in *Drosophila* (Bai et al., 2013; Scherfer et al., 2013), our Lysotracker and Lamp1 staining experiments showed that in healthy adult fruit fly thoracic muscle, there is a minimum level of autophagy, and that this level did not differ much between our control and experimental lines that either reduced or enhanced Activin signaling in muscle. When we starved our control and experimental flies to induce high levels of autophagy in adult muscle, we did not see any changes in the levels of ubiquitinated protein aggregates in adult muscle of our Activin signaling knockdown flies or from the muscle of flies that had enhanced Activin signaling as compared to controls. These data suggest that autophagy may not be the critical mediator of Activin signal-regulated ubiquitinated protein degradation in adult muscle. Once again, the discrepancy between our findings and that of Bai et al. (2013) with respect to the role of autophagy in regulating protein homeostasis in response to Activin signaling will require further study.

In summary, our data suggest that Activin signal reception levels in adult muscle can modulate the adult fly lifespan by directly or indirectly regulating the function of 26S proteasomes. A model depicting our view for how Activin signaling may regulate the lifespan of adult flies is illustrated in Fig. 8F. Because of the conserved nature of Activin signaling across different animal phyla, the observations made in this study may well be extended to other animal species.

## MATERIALS AND METHODS

### Fruit fly genetics, husbandry, and ageing observation

The three transgenic lines used in this study, *Actβ-Gal4*, *Daw-Gal4*, and *Myo-Gal4*, have been described and characterized in fruit fly larvae previously (Awasaki et al., 2011; Kim and O'Connor, 2014; Song et al., 2017a; Zhu et al., 2008). A number of TRiP RNAi lines, which were created in a vermillion (*v^1^*) genetic background, for specific genes in Activin signaling pathway were obtained from Bloomington Stock Center, which include *UAS-Smox RNAi* line (stock number 43138), *UAS-Actβ RNAi* line (stock number 42795), *UAS-Daw RNAi* line (stock number 34974), *UAS-Daw RNAi* line (stock number 50911), and *UAS-Myo RNAi* line (stock number 31200) have been used in our study. Additional fruit fly lines received from Bloomington Stock Center include two *tubGal80^ts^* lines and a fat body specific Gal4 driver line *CG-Gal4* and a number of proteasome RNAi lines such as a *UAS-Prosα6 RNAi* line (stock number 53974), a *UAS-Prosβ5 RNAi* line (stock number 34810), and a *UAS-Rpn1 RNAi* line (stock number 34348). A *UAS-dSmad2 RNAi* line from Vienna RNAi Stock Center was also used, and similarly caused lifespan-shortening effect when expressed in adult fruit fly muscle tissues (data not shown). A *UAS-Babo RNAi* line, *UAS-Babo RNAi^10E2/6E2^*, in which two individual *UAS-Babo RNAi* transgenes were recombined onto the same second chromosome, was created and provided by O'Connor laboratory. Individual transgenic fruit fly lines that were used for expressing wild-type Daw or Myo in this study were also created and provided by O'Connor laboratory, which include *UAS-Daw^9D2^*, *UAS-Daw^9A3^*, *UAS-Myo^4D2^*, and *UAS-Myo^7D3^*. All these transgenic lines were created by cloning individual wild-type *Daw* or *Myo* cDNA into a pUAST vector so that each transgene is under the control of UAS DNA sequences. Individual *pUAST-Daw* or *pUAST-Myo DNA* construct combined with a δ2-3 plasmid that carries a recombinase gene was injected to the pole cells of yellow white (*yw*) fruit fly embryos to get transgenic fly lines.*UAS-Daw^9D2^* transgenic fly line has a *UAS-Daw* transgene inserted onto the second chromosome while *UAS-Daw^9A3^* fly line's *UAS-Daw* transgene was inserted onto the third chromosome in the fruit fly genome. The difference of the two transgenic fruit fly lines *UAS-Myo^4D2^* and *UAS-Myo^7D3^* lies in different insertion sites of the *UAS-Myo* transgene in two different chromosomes. *UAS-Myo^4D2^* has the *UAS-Myo* transgene inserted onto the second chromosome while *UAS-Myo^7D3^* has the *UAS-Myo* transgene inserted onto the third chromosome.

For ageing studies, we crossed different transgenic fly lines to either a *Tub-Gal80^ts^; Tub-Gal4* line or to a *Tub-Gal80^ts^; Mhc-Gal4* line at 18°C, and raised the progeny from these crosses at 29°C for ageing observation. Two RU486 gene switch lines, *TubGS* and *DaGS*, were kindly provided by Dr Marc Tatar and used in our study. RU486 was purchased from Sigma-Aldrich and dissolved in ethanol. Treatment of *DaGS* or *TubGS/UAS-Babo RNAi* or *DaGS or TubGS/UAS-Smox RNAi* fruit flies with mifepristone (RU486 from Sigma-Aldrich) at a final concentration of 50 µg/ml in fruit fly food was performed by following a standard protocol (Poirier et al., 2008). For ageing observation, we collected newly hatched male and female flies for each genotype and put them separate food vials. On average, each vial contains about 15 to 20 fruit flies. The survivorship of both control and experimental groups of fruit flies was monitored either daily or every other day until the whole data recording was done. During data recording time, the fruit flies in any vials that showed any dryness of the food were immediately transferred to fresh vials.

All of our flies were raised on standard corn meal, which was made based on a recipe from Bloomington Fruit Fly Stock Center with minor modifications. For each batch of fly food, we cooked 730 g of yellow cornmeal, 100 g of soy flour, 173 g of dry yeast, 769 ml of corn syrup, and 58 g of agar in 10 liters of tap water. After the food was cooked, we added 67.5 ml propionic acid (from Sigma-Aldrich) and 66 ml of 30% methylparaben (tegosept) stock solution, which was made by dissolving methylparaben in 200 proof (≥99.5%) ethanol, to the food as anti-microbe reagents. The fruit flies experienced daily 12-h light and 12-h dark cycles. Humidity levels were kept at about 40%.

### Western blot

Phospho-Smox (P-Smox) proteins and a 26S proteasome subunit (IIG7) protein were detected from larval or adult thoracic muscle tissues by using an anti-P-Smox primary antibody (Cell Signaling Technology) and a mouse monoclonal antibody (IIG7) against a proteasome catalytic α subunit (Santa Cruz Biotechnology), respectively, on western blots. For a loading control, an antibody against fruit fly β-tubulin (Developmental Hybridoma Bank) was used. For western blots, thoracic tissues from three or five adult fruit flies were homogenized in 18 μl RIPA buffer (150 mM NaCl, 1.0% IGEPAL^®^ CA-630, 0.5% sodium deoxycholate, 0.1% SDS, 50 mM Tris, pH 8.0) from Sigma-Aldrich supplemented with 3 μl 7× protease inhibitor on ice. Homogenized tissue lysates were centrifuged at 13,000 ***g*** at 4°C for 15 min before the supernatant was taken and mixed with a sodium dodecyl sulfate (SDS) page gel loading buffer. The prepared protein extracts containing loading buffer were separated on commercial available SDS page gels by standard electrophoresis. After electrophoresis, proteins from SDS page gel were transferred to a polyvinyl difluoride (PVDF) membrane. Diluted primary antibody was incubated with PVDF membrane at 4°C overnight. After washing the membrane with washing buffer three times, the membrane was incubated with horse radish peroxidase (HRP)-conjugated secondary antibody at room temperature for 1 h before the final wash. Western blot signals were developed using a standard chemiluminescent kit. After scanning the signals for P-Smox proteins, the PVDF membrane were striped and re-incubated with an anti-β-tubulin primary antibody. The protein bands detected by anti-β-tubulin antibody were used to quantify the loading of the total proteins of each lane. The relative levels of P-Smox proteins or proteasome α subunit proteins from each lane was quantified by the protein levels of β-tubulin from the same lane in which P-Smox proteins or proteasome α subunit proteins were detected. ImageJ (NIH) image quantification software was used to determine relative protein levels.

### Immunohistochemistry and confocal microscope imaging

For immunostaining of adult fruit fly muscle tissues, the thoracic muscle was dissected in phosphate buffered saline solution (PBS) at room temperature using a razor blade and fine tip forceps. Dissected tissues were fixed in 3.7% formaldehyde in PBS at room temperature for 30 to 60 min. Fixed tissue was washed three times (5 to 10 min each time) in PBT solution (0.1% triton X-100 in PBS) before diluted primary antibody in PBT was added to the tissue. The incubation of the primary antibody with the tissue was done either at room temperature for 2 h or at 4°C overnight on a rocking platform. Following incubation of the tissue with a primary antibody, the tissue was washed again three times in PBT. The PBT washed tissues were incubated with diluted secondary antibody (1:200 in PBT) at room temperature for 1 to 2 h before the tissues were washed again three times in PBT solution and mounted in a 50% glycerol /50% PBS solution for imaging. Both primary and secondary antibodies were diluted in PBT solution. The dilution factor for mouse anti-mono- and polyubiquitinated monoclonal antibody (FK2) (Enzo Life Sciences, Farmingdale, USA) was 1:200. Rabbit anti-fruit fly LAMP1 antibody was purchased from Abcam and diluted in PBT with a dilution factor of 1:100 for the immunostaining. The secondary antibody Alexa Fluor 594 goat anti-mouse IgG (H+L) purchased from Molecular Probes (Eugene, USA) was also diluted in PBT with a dilution factor of 1:200. Muscle fibers were stained by a CytoPainter Phalloidin-iFluor 488 reagent (1:1000 dilution) from Abcam for F-actin molecules. A confocal microscope and a fluorescence compound microscope (Nikon, New York, USA) were used for imaging.

### Lysotracker staining of autophagosomes

Lysotracker solution purchased from Abcam was diluted as recommended in the Live Cell Solution coming from the Lysotracker kit. Fifty microliters (50 μl) of the diluted Lysotracker staining solution was dispensed onto fresh fly food in vials. Flies to be tested were transferred into these vials for a period of 24 h. Subsequently, those flies fed Lysotracker were anesthetized with CO_2_, and dissected in PBS. The thorax was isolated and sliced longitudinally to produce four sections. The samples were then fixed in 4% formaldehyde PBS fixing solution for 30 min. The tissues were then washed two times in PBS for 30 min. After being washed in PBS solution, the tissues were incubated for 2 h at room temperature with CytoPainter Phalloidin iFluor 488 F-actin stain (Abcam) diluted in PBS with a dilution factor of 1:1000 to counter stain the muscle fibers. Following F-actin staining, the tissues were then washed two times for 30 min at room temperature. At the end, our Lysotracker and F-actin stained muscle tissues were mounted on slides in anti-fade mounting solution (Sigma-Aldrich), imaged by using a fluorescence compound microscope and analyzed with ImageJ software.

### Data analysis

Ageing data were analyzed with an online ageing data analysis tool called OASIS (Yang et al., 2011). Statistical methods of Fisher's exact test and Log-rank test were used for ageing data analysis with the OASIS online tool. The Lysotracker staining results were analyzed using an Anova statistical method. Protein aggregates and autophagosomes were quantified by ImageJ software. The protein levels of P-Smox or the proteasome α subunit were analyzed and quantified by Gel Analyzer software (http://www.gelanalyzer.com/index.html).

## Supplementary Material

Supplementary information

First Person interview
