# Supplementary material for: The Drosophila TGF-beta/Activin-like ligands Dawdle and Myoglianin appear to modulate adult lifespan through regulation of 26S proteasome function in adult muscle
Source: Biol Open. 2018 Apr 3;7(4):bio029454. doi: 10.1242/bio.029454 (PMC5936056; doi:10.1242/bio.029454)
Supplement: Supplementary information [file biolopen-7-029454-s1.pdf]

© 2018. Published by The Company of Biologists Ltd | Biology Open (2018): doi:10.1242/bio.029454: Supplementary information

FIRST PERSON

First person – Shaughna Langerak

First Person is a series of interviews with the first authors of a selection of papers published in Biology Open, helping early-career researchers promote themselves alongside their papers. Shaughna Langerak is first author on 'The *Drosophila* TGF-beta/Activin-like ligands Dawdle and Myoglianin appear to modulate adult lifespan through regulation of 26S proteasome function in adult muscle', published in BiO. Shaughna is a MSc student in the lab of Jennifer Schisa at Central Michigan University, USA, using *C. elegans* to investigate endoplasmic reticulum remodeling and ribonucleoprotein assembly in oocytes and how remodeling affects oocyte quality.

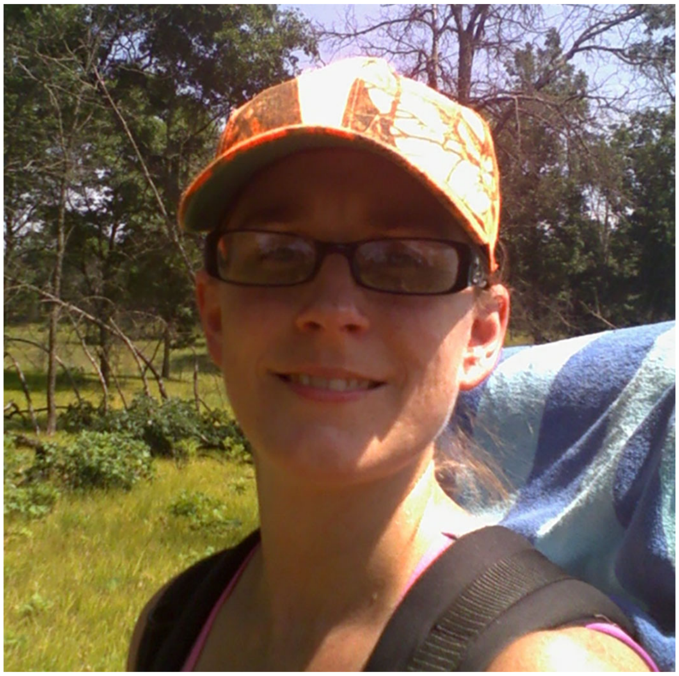

Shaughna Langerak

What is your scientific background and the general focus of your lab?

I completed my undergrad with dual biology degrees with a concentration in environmental biology and another in biotechnology at Ferris State University. I chose to combine these programs because I felt that it was important to explore multiple approaches to scientific inquiry. I became interested in research early in my academic career and joined a lab my freshman year in college. In general my research focus has always been exploring ageing and cellular remodeling. During my early research I worked with fruit flies investigating cell signaling and lifespan and currently I am working with *C. elegans* investigating cellular remodeling.

> "I became interested in research early in my academic career and joined a lab my freshman year in college."

How would you explain the main findings of your paper to non-scientific family and friends?

Our main findings are that in muscle tissue a cell signaling pathway called Activin plays a role in regulating 'machinery' called proteasomes, which are responsible for the removal of 'waste' proteins. When this pathway was turned up, the flies in our experiments lived longer and when this system was turned down the flies had shorter life spans. Because humans also have a version of this pathway our findings may translate to, or help find, new therapies to preserve or prolong health and function in older individuals.

What are the potential implications of these results for your field of research?

Ubiquitination of damaged or misfolded proteins and their ensuing degradation through proteasomes is a well-conserved cellular mechanism required for maintaining normal protein homeostasis in healthy tissues; however, this activity declines as organisms age. How the decline of proteasome function takes place and what factors are responsible for the regulation of levels of proteasome components are not fully understood. Our data suggest that Activin signaling initiated by the ligands Myo and Daw in adult *Drosophila* muscles influences protein homeostasis through either direct or indirect regulation of 26S proteasome levels. Since Myo is closely related to the vertebrate muscle mass regulator Myostatin (GDF8) and the Myostatin paralog GDF11, our findings may offer a new experimental model for investigating the roles of GDF11/8 in ageing regulation in vertebrates.

What has surprised you the most while conducting your research?

The thing that surprised me the most during my research is how often small details in the outcomes of experiments can turn your research focus in an unexpected direction. For instance, during my research exploring Activin signaling and ageing I noticed that when the Activin receptor Babo was downregulated the flies displayed elevated wings, which is a weakened muscle phenotype. Before that we had no indication that the life span-modulating effects that we observed were due to changes in muscle tissue health. We then began investigating the cause of this phenotype, we looked at mitochondrial function and then protein homeostasis. After determining that the cause was loss in protein homeostasis we next explored the roles of autophagy and 26S proteasome activity in our results. It was serendipitous that I noted the slight change in the experimental flies' wing posture but that small detail enlightened our research and was a key clue as to how modulation of the Activin pathway affects *Drosophila* life span.

Shaughna Langerak's contact details: Biosciences 2100, Central Michigan University, Mount Pleasant, MI 48859, USA.
E-mail: lange1sh@cmich.edu

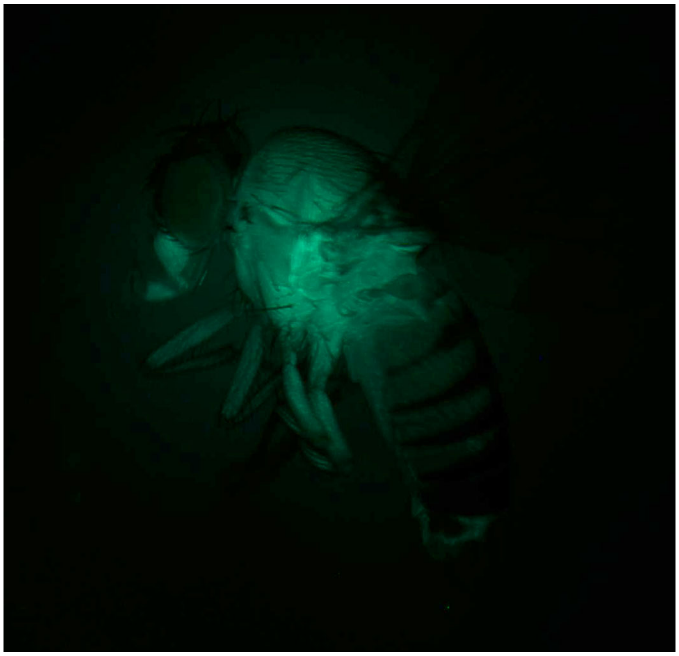

Adult fruit fly with GFP expression driven by the muscle tissue-specific driver Mhc-Gal4.

What, in your opinion, are some of the greatest achievements in your field and how has this influenced your research?
I believe that the greatest achievements in the field of biology have been the development of genetic tools that allow researchers to explore even the most difficult questions. RNAi, CRISPR/CAS9, UAS/GAL4 – in recent decades and years the list of options has exploded and will most likely continue. By having the ability to modify gene expression and control those changes temporally and spatially the opportunities to explore seem only limited by your imagination. In my research the ability to use these tools has allowed me to really ask the questions I wanted to ask through experimentation and added to the robustness of the answers by allowing me to use multiple approaches.

"I believe that the greatest achievements in the field of biology have been the development of genetic tools that allow researchers to explore even the most difficult questions."

What changes do you think could improve the professional lives of early-career scientists?
I think that early-career scientists would benefit from early education in communicating with the general public alongside learning professional scientific communication methods. The entire scientific community and the public would benefit from a focus on tailoring a more robust and direct method of informing the public of findings. There is a disconnect between communication within the scientific community and with how that is relayed to the general population. This issue continues to be pervasive even though much of the funding the scientific community relies on requires the interest of average citizens. By not crossing the chasm between the two so much is lost from financial support to simple confidence.

What's next for you?
I absolutely love research and will most likely continue in some capacity but my immediate plans for the future are to transition to a science teacher in secondary education. I feel that I can have the greatest impact in the scientific community by stepping into a role where I can reach young people with my enthusiasm and love of science and research and hopefully influence ideas about who does science, what it's all about, the opportunities that exist and how to really think critically.

Reference
Langerak, S., Kim, M. -J., Lamberg, H., Godinez, M., Main, M., Winslow, L., O'Connor, M. B. and Zhu, C. C. (2018). The *Drosophila* TGF-beta/Activin-like ligands Dawdle and Myoglianin appear to modulate adult lifespan through regulation of 26S proteasome function in adult muscle. *Biol. Open* **7**: bio029454, doi:10.1242/bio.029454.
